# Supplementary material for: Synergistic Effect of Motivation for the Elderly and Support for Going Out II: Measures to Induce Elderly Men to Go Out
Source: Geriatrics (Basel). 2024 Feb 18;9(1):21. doi: 10.3390/geriatrics9010021 (PMC11285032; doi:10.3390/geriatrics9010021)
Supplement: Supplementary file 1 [file geriatrics-09-00021-s001.zip › geriatrics-2739624-supplementary.pdf]

Supplementary Table S1. Changes in each variable before and after Choisoko system use.

	Men Group (N = 16)		Women Group (N = 47)		
**Valuable**	**N**	**Mean ± SD**	**N**	**Mean ± SD**	***p*-value**
Height	10	−2.6 ± 5.9	39	0.4 ± 2.2	0.151
Weight	10	−1.4 ± 7.3	37	0.2 ± 1.4	0.508
BMI	10	0.2 ± 3.2	37	0.0 ± 1.0	0.820
Body fat percentage	10	2.2 ± 8.9	35	0.2 ± 2.5	0.502
Alb	10	−0.1 ± 0.2	40	−0.1 ± 0.2	0.944
AST	10	5.1 ± 14.2	40	−0.6 ± 7.0	0.245
ALT	10	−1.1 ± 6.5	40	−1.0 ± 5.8	0.957
BUN	10	1.8 ± 4.8	40	1.3 ± 4.2	0.776
CRE	10	−0.1 ± 0.2	40	0.0 ± 0.1	0.404
eGFR	10	1.7 ± 5.9	39	1.7 ± 6.3	0.994
WBC	10	−290.0 ± 528.0	40	202.5 ± 1468.7	0.093
RBC	10	−7.4 ± 22.0	40	−15.4 ± 18.1	0.312
Hb	10	0.1 ± 0.6	40	−0.2 ± 0.6	0.188
Ht	10	−0.3 ± 2.1	40	−0.8 ± 1.7	0.481
MCV	10	0.8 ± 1.5	40	1.6 ± 1.7	0.184
MCH	10	0.8 ± 0.7	40	0.8 ± 0.9	0.804
MCHC	10	0.5 ± 0.8	40	0.3 ± 0.9	0.453
Platelet	10	−0.2 ± 2.4	40	−0.2 ± 2.7	0.953
Grip strength (right)	10	−0.7 ± 2.0	38	−1.3 ± 3.0	0.446
Grip strength (left)	10	−2.0 ± 3.6	38	−1.4 ± 2.2	0.678
Stand up	9	−0.9 ± 3.1	37	1.3 ± 12.9	0.357
Walk speed	10	0.1 ± 0.5	38	0.1 ± 1.8	0.950
Walk speed max	10	−3.1 ± 11.0	38	−0.3 ± 2.3	0.448
Muscle	10	−1.4 ± 2.6	35	0.1 ± 0.9	0.111
Volume of iliopsoas muscle	10	−3630.0 ± 14140.2	40	−470.0 ± 6605.3	0.507
FIM total score	10	0.6 ± 1.3	40	0.0 ± 0.8	0.204
FIM exercise score	10	0.4 ± 0.8	40	0.0 ± 0.5	0.155
FIM cognitive score	10	0.2 ± 0.6	40	0.0 ± 0.4	0.424
